# The Antidiabetic Mechanisms of Polyphenols Related to Increased Glucagon-Like Peptide-1 (GLP1) and Insulin Signaling

**DOI:** 10.3390/molecules22060903

**Published:** 2017-05-30

**Authors:** J. Abraham Domínguez Avila, Joaquín Rodrigo García, Gustavo A. González Aguilar, Laura A. de la Rosa

**Affiliations:** 1 Coordinación de Tecnología de Alimentos de Origen Vegetal, Centro de Investigación en Alimentación y Desarrollo A. C., Carretera a La Victoria km 0.6, AP 1735, Hermosillo 83304, Sonora, Mexico; abrahamdominguez9@yahoo.com, (J.A.D.A.); gustavo@ciad.mx, (G.A.G.A.); 2 Departamento de Ciencias de la Salud, Instituto de Ciencias Biomédicas, Universidad Autónoma de Ciudad Juárez, Anillo Envolvente del PRONAF y Estocolmo s/n, Ciudad Juárez 32310, Chihuahua, Mexico; jogarcia@uacj.mx; 3 Departamento de Ciencias Químico-Biológicas, Instituto de Ciencias Biomédicas, Universidad Autónoma de Ciudad Juárez, Anillo Envolvente del PRONAF y Estocolmo s/n, Ciudad Juárez 32310, Chihuahua, Mexico

**Keywords:** beta cells, diabetes, insulin, DPP4 inhibition, GLP1, L cells, pancreas, polyphenols

## Abstract

Type-2 diabetes mellitus (T2DM) is an endocrine disease related to impaired/absent insulin signaling. Dietary habits can either promote or mitigate the onset and severity of T2DM. Diets rich in fruits and vegetables have been correlated with a decreased incidence of T2DM, apparently due to their high polyphenol content. Polyphenols are compounds of plant origin with several documented bioactivities related to health promotion. The present review describes the antidiabetic effects of polyphenols, specifically related to the secretion and effects of insulin and glucagon-like peptide 1 (GLP1), an enteric hormone that stimulates postprandial insulin secretion. The evidence suggests that polyphenols from various sources stimulate L-cells to secrete GLP1, increase its half-life by inhibiting dipeptidyl peptidase-4 (DPP4), stimulate β-cells to secrete insulin and stimulate the peripheral response to insulin, increasing the overall effects of the GLP1-insulin axis. The glucose-lowering potential of polyphenols has been evidenced in various acute and chronic models of healthy and diabetic organisms. Some polyphenols appear to exert their effects similarly to pharmaceutical antidiabetics; thus, rigorous clinical trials are needed to fully validate this claim. The broad diversity of polyphenols has not allowed for entirely describing their mechanisms of action, but the evidence advocates for their regular consumption.

## 1. Introduction

Type-2 diabetes mellitus (T2DM) is a non-communicable disease that is exceedingly common in modern societies. Its formal diagnostic criteria are: (1) glycated hemoglobin (HbA1c) concentration ≥ 6.5%, or (2) fasting glycemia ≥ 126 mg/dL (7.0 mM), or (3) glycemia ≥ 200 mg/dL (11.1 mM) two hours after an oral glucose load [1]. Projections indicate that the global incidence of diabetes will continue to increase from 2010 to 2030, and some authors project 439 million adult patients by 2030 [2], while others predict 552 million by 2030 [3]. According to a 2015 report, 422 million people already had diabetes by 2014 [4], which suggests that the predicted number of cases may be exceeded. T2DM is a degenerative disease that progressively decreases optimal function of the cardiovascular system, eyes, kidneys, nervous system and other organs such as the skin, liver and gut. This is reflected on a reduced quality of life of the patient and their immediate family members or caregivers, and an economic burden on the workforce and the health system [5]. The symptoms of diabetes have been known since ancient times, but a thorough understanding of its underlying causes and effective treatments were not fully achieved until the 20th century [6]. Being a disease of affluence, it was not as prevalent as compared to current times; changes in dietary habits and an increasingly sedentary lifestyle have been continuously referenced as main culprits for its increase. Long-term consumption of diets rich in simple carbohydrates (such as high-fructose corn syrup), lipids (particularly *trans* and saturated lipids), and energy, and low in fiber and micronutrients are associated with obesity and T2DM [7].

On the contrary, some dietary choices can elicit positive health effects by preventing T2DM, delaying age of onset, or mitigating its symptoms. A prime example is the Mediterranean diet (rich in fruits and vegetables and low in simple carbohydrates and processed foods), which is often linked with such benefits [8]. Complementary studies have shown that polyphenols contained in fruits and vegetables are key mediators of the antidiabetic effects attributed to them. Polyphenols are secondary metabolites of plant origin that are synthesized from l-phenylalanine or l-tyrosine through the phenylpropanoid pathway [9]. They have at least one phenolic moiety as part of their main molecular skeleton, and their complexity and structural diversity extends from simple phenolic acids to highly complex polymerized tannins. The covalent attachment of monomeric/oligomeric carbohydrates further extends their structural and functional diversity. Polyphenols are a highly active research discipline due to their ubiquity in edible plants and the accumulated data obtained in the last decades, which shows that they have striking effects on different aspects of human health. They exert anti-inflammatory, anti-proliferative and anti-oxidant activities [10], in addition to the previously stated antidiabetic effects.

Because diabetes is multifactorial, the antidiabetic effects of polyphenols are also multifactorial. Polyphenols can modulate the digestion of starch and other carbohydrates [11], they induce satiety [12], mitigate non-enzymatic glycation, modulate hormonal responses [13], among several others, which are altogether antidiabetic actions. In this work, we are interested in the effects that polyphenols have on the secretion and signaling of glucagon-like peptide-1 (GLP1) and insulin, two hormones whose insufficient or inappropriate activity ultimately leads to diabetes. Since GLP1 stimulates the secretion of insulin (and prevents the release of glucagon), their effects are physiologically intertwined. They are also main molecular targets of antidiabetic medications, which highlights their role in all aspects of T2DM.

## 2. GLP1 and Insulin Signaling

Glycemic control is a tightly coordinated process that requires precise glucose sensing and adequate endocrine response of the pancreas, along with a corresponding response from peripheral tissues. This section briefly describes the normal synthesis, secretion and signaling pertaining to GLP1 and insulin as part of the glucose-lowering response in healthy individuals. Pharmacological options aimed at preserving their function will also be summarized.

### 2.1. GLP1

The human *GCG* gene (2q24.2, Genbank: 2641) codes for proglucagon, a 160 aminoacid peptide expressed in the pancreas, gut, and brain, that is post-translationally processed into different peptides according to the organ in question. In L-cells of the small intestine, proglucagon is cleaved into active GLP1 (and other fragments) by prohormone convertase. There is basal serum GLP1 concentration, but its release is mainly postprandially in response to nutrient loads [14]. The GLP1 receptor (GLP1R) is a 7-transmembrane-spanning heterotrimeric G-protein-coupled receptor (GPCR). It is expressed on various cell types, including pancreatic β-cells; here, intracellular signaling takes place through cAMP and other second messengers upon GLP1 binding, which promotes insulin secretion and gene transcription [15]. GLP1 and the related glucose-dependent insulinotropic polypeptide (GIP, also known as gastric inhibitory polypeptide) promote insulin secretion and other effects, such as hypothalamic stimulation to induce postprandial satiety. GLP1 and GIP are both known as incretins, and their effects are considered antidiabetic.

Secreted GLP1 has a very short serum half-life of approximately two minutes, due to extensive hydrolysis by dipeptidyl peptidase-4 (DPP4). DPP4 is located on the surface of the endothelium, but it can also be found in a soluble circulating form (sDPP4). It is a serine protease capable of hydrolyzing several substrates, particularly those with a proline or alanine residue at position 2, relative to its amino terminus. In addition to GLP1, erythropoietin, growth hormone-releasing hormone, neuropeptide Y, peptide tyrosine-tyrosine (PYY), and others are well known DPP4 substrates, which highlight the numerous processes that are influenced by its activity. Its effects have been extensively analyzed, both on GLP1 and on its other targets [16].

### 2.2. Insulin

Pancreatic β-cells translate the *INS* gene (11p15.5, Genbank: 3630) into an immature peptide named preproinsulin that is 110 aminoacids long. Preproinsulin is translocated into the endoplasmic reticulum (ER), where a signal peptidase cleaves the signal peptide, which forms the still immature proinsulin. One intrachain and two interchain disulfide bonds are formed within the ER, which also promotes near-final folding. Proinsulin dimerizes, complexes with Zn^+2^ ions and enters the Golgi apparatus, where prohormone convertases and carboxypeptidase E cleave proinsulin into mature insulin and C-peptide (31 aminoacids) [17,18]. The resulting insulin produced after these events is a peptide hormone that consists of an A chain and a B chain of 21 and 30 aminoacids in length, respectively, linked together by the disulfide bonds that were previously formed. β-cells secrete insulin in response to increased glycemia and by GLP1 stimulation (as previously stated). Its main target tissues are skeletal muscle, adipose tissue, and liver, where the insulin receptor (IR) is highly expressed [19].

The IR is a tyrosine kinase receptor; its structure consists of two extracellular α subunits and two transmembrane β subunits linked by disulfide bonds. The binding of insulin initiates a homodimerization of the IR, conformational changes and a phosphorylation cascade. Initial phosphorylation takes place on the receptor itself, and then on the insulin receptor substrates (IRS). Phosphorylated IRS recruit other downstream proteins that exert various effects on the cell, through the phosphatidylinositol triphosphate (IP3), Ras and other pathways [19]. One of these effects is the fusion of vesicles to the cell membrane that contain stored glucose transporter 4 (GLUT4), and, ultimately, the signaling cascade concludes with the incorporation of GLUT4 into the cell membrane, allowing glucose to passively diffuse through it. The net physiological outcome is an incorporation of glucose into tissues and preservation of euglycemia.

Insulin resistance is characterized by a progressive decline of the normal response to the hormone; a state of hyperinsulinemia results from the pancreas increasing its secretion in an effort to counter hyperglycemia. The pancreas will eventually fail to maintain glucose levels within the physiological range, at which time the diagnostic criteria for diabetes will be met and the disease will be diagnosable by the previously mentioned criteria. Interestingly, when there is insulin resistance, the secretion of incretins is not increased, but β-cells become more responsive to them, and oral nutrient loads exert a stronger insulin response because of this phenomenon [20].

### 2.3. Current Antidiabetics that Target Insulin or GLP1

There are numerous currently available antidiabetic treatment options with different mechanisms of action. In order to compare the antidiabetic effects of polyphenols to pharmaceuticals, this section provides a brief overview, and only those related to GLP1 or insulin are considered.

1. Gliptins are DPP4 inhibitors that prevent DPP4-mediated GLP1 hydrolysis. Sitagliptin was the first to be approved by the United States Food and Drug Administration (FDA) in 2006, while vildagliptin, saxagliptin and linagliptin were subsequently approved; this makes them the most recently launched antidiabetics. Gliptins exert an 80–97% effective DPP4 inhibition through a reversible-competitive mechanism, yielding an increased GLP1 half-life of up to 5 min, in contrast with its physiological half-life of less than 2 min [21,22]. Increased GLP1 half-life allows additional β-cell stimulation and ensuing insulin release.

2. Incretin mimetics are chemically modified GLP1 molecules that act as synthetic agonists of the GLP1R. They mimic GLP1 signaling on β-cells, which amplifies physiological GLP1-mediated insulin secretion. They were developed because administration of GLP1 is impractical due to its short half-life, and would otherwise require continuous intravenous administration, while analogues are administered subcutaneously. Some analogues (exenatide and lixisenatide) have a different aminoacid sequence to human GLP1, which results in a decreased affinity of DPP4 towards them, and therefore a longer half-life. Others (liraglutide) have a covalently-attached fatty acid that increase their binding to albumin and minimize renal filtration, while yet another strategy is to covalently bind the analogue to albumin (albiglutide) or to the Fc fragment of IgG (dulaglutide) [23].

3. Insulin secretagogues stimulate the pancreas to produce and secrete insulin, and are subclassified as sulfonylureas or glinides. Sulfonylureas have been commercially available since the 1950s, and glibenclamide is currently the most representative member of this class, along with glimepiride and others. Sulfonylureas bind to the sulfonylurea receptor 1 (SUR1) and inhibit ATP-sensitive potassium channels (K_ATP_) localized to the cellular membrane of pancreatic β-cells, which results in membrane depolarization, increased intracellular calcium concentration and ultimately, insulin secretion [24]. Glinides have a similar mechanism of action to sulfonylureas, which rely on K_ATP_ channel inhibition, calcium influx and subsequent insulin release, but their molecular structure, binding site, and duration of action (more rapid and shorter than sulfonylureas) allows clear distinction between them [25]. They are considered safe in most cases, but one of their main and most serious side effects is hypoglycemia [26]. It should be noted that gliptins, incretin mimetics, sulfonylureas, and glinides are only pharmacologically active when administered to patients with some β-cell activity and cease to be effective once there are no functioning β-cells that are able to produce insulin.

4. Another strategy to treat T2DM is to increase peripheral sensitivity to insulin by thiazolidinediones (TZDs, also known as glitazones), named according to their molecular structure. TZDs are synthetic agonists of the peroxisome proliferator-activated receptor gamma (PPARγ) transcription factor that is expressed in the liver, skeletal muscle, and adipose tissue. Endogenous PPARγ ligands are mainly long chain omega-3 fatty acids. Active PPARγ modulates the transcription of genes related to insulin sensitivity and metabolism of carbohydrates and lipids [27].

**Figure 1:**
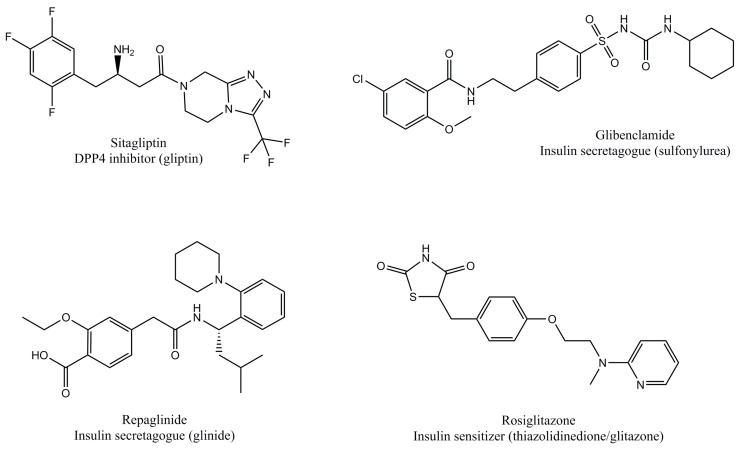
Molecular structures of representative pharmacological antidiabetics listed in Table 1. The peptide structure of glucagon-like peptide-1 receptor (GLP1R) agonists was omitted for clarity.

**Figure 2:**
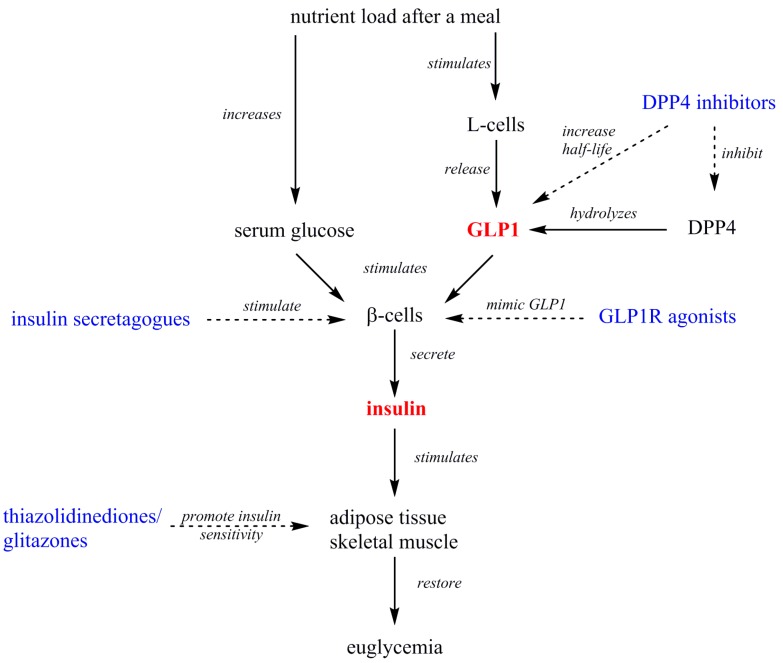
Overview of the events that take place after a meal that lead to GLP1 and insulin secretion. The different pharmacological antidiabetics are highlighted in blue, and their actions are indicated by dashed arrows.

These pharmacological treatments can be prescribed as monotherapy or in combination, and often other kinds of drugs are also concomitantly prescribed, such as statins, ezetimibe, α-glycosidase inhibitors, and others. Table 1 summarizes the previously described antidiabetic compounds, and Figure 1 illustrates the molecular structure of representative members. Figure 2 illustrates the combined signaling of GLP1 and insulin at the whole organism-level, and the site of action of antidiabetics.

## 3. Effects of Polyphenols on GLP1 and Insulin Signaling

**Figure 3:**
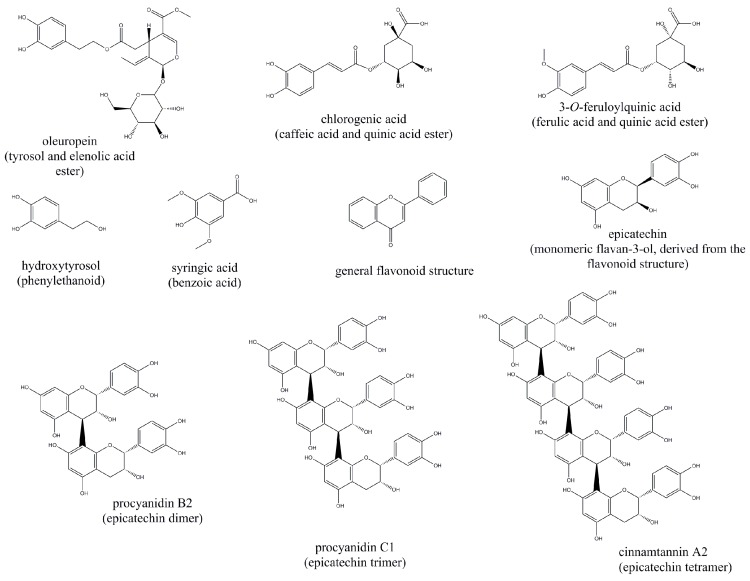
Molecular structures of the polyphenols with potential antidiabetic effects discussed in the main text.

GLP1, insulin and the peripheral actions of insulin are strongly influenced by dietary components. Macronutrients are the most notable examples, but polyphenols and other micronutrients have also been documented as important regulators. The structures of the molecules discussed hereafter are depicted in Figure 3.

### 3.1. Effects of Polyphenols on GLP1

Nagamine et al. [28] prepared an ethanolic extract of sweet potato (*Ipomoea batatas* L.) cv Suioh, rich in the following derivatives of caffeoylquinic acid (CQA): 3-CQA, 3,4-diCQA, 3,5-diCQA, 4,5-diCQA, and 3,4,5-triCQA. Murine GLUTag cells (a GLP1-secreting enteroendocrine cell line) were incubated with either the sweet potato extract (10 mg/mL), the individual CQA derivatives previously mentioned (10 mM each), glutamine as positive control (10 mM) or dimethyl sulfoxide (DMSO, 0.1%) for 2 h. GLP1 was quantified after the incubation period by ELISA. Results showed that all analyzed compounds increased GLP1 secretion, but the most prominent effect was induced by 3,4,5-triCQA (~650 pM), followed by the sweet potato extract (~500 pM) as compared to the control (~50 pM) and the positive control (~150 pM). It is apparent from these results that the most effective stimulator of GLP1 secretion is the tri-CQA derivative, but interestingly, it is the least abundant component of the extract (~1.1%). Follow-up experiments by the authors corroborated their results on male Sprague–Dawley rats, by demonstrating that a 2 g/kg of body weight (BW) orally administered (p.o.) dose of the sweet potato extract stimulated GLP1 secretion after an intraperitoneal (i.p.) glucose load (2 g/kg·BW), and mitigated the subsequent hyperglycemia. The i.p. glucose dose was higher than the normally administered dose (1.75 mg/kg) in oral glucose tolerance tests. These results suggest that the polyphenolic components of sweet potato exert antidiabetic effects, particularly tri-CQA, but the mechanism of action on L-cells was not investigated.

Coffee is a widely consumed beverage with high phenolic content, mainly chlorogenic acid, which has a potential GLP1 secretagogue ability. Fujii et al. [29] prepared a caffeine-free coffee polyphenol extract (CPE) whose main components were mono-, di-CQA, and feruloylquinic acid (FQA) derivatives. Human NCI-H716 cells (GLP1-secreting cells from colorectal adenocarcinoma) were incubated for 2 h with CPE in increasing concentrations (0, 0.01, 0.05 or 0.1%) or with phorbol 12-myristate 13-acetate (12 μM) as positive control. For comparison, a coffee grain extract can contain up to 0.045 *w/w* of chlorogenic acid isomers [30]. The CPE exerted a dose-related increase in GLP1 secretion, which was likely related to an increase in intracellular cAMP (cAMP_i_), which followed a similar trend. To determine the effects of the CPE on an in vivo model, male C57BL/6J mice were given CPE (0.6 g/kg·BW, p.o.), which resulted in increased portal GLP1 concentration after the nutrient load (quantified after 10 and 30 min), but without an effect on glycemia, GIP, or insulin concentration. When the CPE was co-administered with glucose, glycemia decreased faster in the CPE-treated mice. These experiments suggest that coffee CQA and FQA derivatives can stimulate GLP1 secretion in a cell line and in an in vivo model, possibly through an increase in cAMP_i_ concentration. The authors suggest that GPCRs may be the targets of the active compounds, but this was not completely demonstrated.

Cocoa and products derived from it contain flavanols as their main polyphenolic constituents. Ryan et al. [31] compared the in vitro DPP4 inhibition potential of cocoa products with different processing methods. Extracts were prepared from cocoa beans, fermented cocoa beans, roasted cocoa liquor, and fermented roasted cocoa liquor, and their DPP4 inhibition ability was compared with diprotin A, a peptide DPP4 inhibitor (Ile-Pro-Ile), as a positive control. They showed that the cocoa polyphenols exert a modest DPP4 inhibition, as compared to the control, and that fermentation and roasting can improve this result. Because the concentration of no individual compound analyzed (catechin, epicatechin, and their oligomers and polymers) showed strong correlation with DPP4 inhibition, they proposed that inhibition may occur through non-specific protein binding or precipitation, and that the melanoidins formed during the roasting process (as products of the Maillard reaction) may enhance protein binding. Although the data suggests that these cocoa products may exert antidiabetic effects, its effect on GLP1 was not directly analyzed.

An anthocyanin-rich grape seed extract (GSE) was used by González-Abuín et al. [32] to analyze its effect on GLP1 secretion in rats as compared to vildagliptin. Three groups were administered p.o. doses of GSE (1 g/kg·BW), vildagliptin (1 mg/kg·BW, positive control) or water (negative control). Two g/kg·BW of glucose was administered 40 min after the treatments, and the animals were sacrificed after an additional 20 min. Results showed that the GSE treatment was as effective as vildagliptin in stimulating GLP1 and insulin secretion and in mitigating hyperglycemia. The observed GLP1 increase was dependent on the presence of glucose, because the GSE had no effect on the glycemia of animals who did not ingest glucose. The effects of the GSE were due to at least two mechanisms, an increase in GLP1 secretion, and its decreased hydrolysis by intestinal DPP4, which the GSE was able to inhibit. The mixture of catechin, epicatechin, procyanidin B2, and gallic acid (all contained in the extract) were able to inhibit DPP4 activity, and all but epicatechin exerted significant individual DPP4 inhibition. It would be interesting to determine if co-administration of vildagliptin and the GSE would synergize on GLP1 secretion or on DPP4 inhibition.

Additional evidence reported by González-Albuín et al. [33] described the effects of GSE on GLP1 secretion from STC-1 cells (secretin tumor cells), an enteroendocrine cell line derived from a double-transgenic mouse tumor. The cells were incubated with 0.05, 0.5, 5, or 50 mg GSE/L of cell medium for 3 h with either glucose, fatty acids (linoleic acid) or aminoacids (proline) to stimulate GLP1 secretion in response to different macronutrients. An ELISA analysis was used to quantify GLP1. They showed that the GSE dose affected cell membrane potential: depolarization was induced by the 0.05 and 0.5 mg GSE/L doses, the 5 mg GSE/L dose had no effect, and the 50 mg GSE/L dose caused hyperpolarization. The highest GSE dose significantly decreased GLP1 secretion, but the others showed no effect, which is contrary to the expected results, particularly when considering the effects described in the in vivo rat model. The direct involvement of mitochondrial glucose metabolism on Na^+^ concentration was likely not responsible for the documented changes in membrane polarization (but is reported in β-cells), and the authors further warn that direct extrapolation of changes in membrane potential to GLP1 secretion may not be accurate. It is also noteworthy that DPP4 inhibition was not determined in this experiment; if DPP4 inhibition were to take place, it could lead to an increase in GLP1 concentration without directly increasing its secretion.

Serrano et al. [34] provided evidence of the acute effects of GSE on GLP1 secretion in rats. Wistar rats were administered GSE (846 mg/kg·BW of phenolics) and an intragastric meal. Twenty minutes after the meal, portal GLP1 concentration was significantly increased by the GSE, in parallel with insulin concentration. The effects on GLP1 were not mediated by changes in mRNA expression of the proglucagon gene, suggesting an increased protein secretion or half-life as the mechanism of action. Additional experiments demonstrated that the GSE exerted a satiety effect related to GLP1 secretion. A structure-functional association was postulated based on the evidence, where the active polyphenolic components are those that bear a galloyl moiety, because those that lack it were not capable of inducing the same actions.

*Hibiscus sabdariffa* is consumed in Africa, Asia and the Americas, particularly Mexico, and can contain up to 121 mg of polyphenols/g of dry matter [35]. Peng et al. [36] used *H. sabdariffa* as a polyphenol source, and administered it to immortalized human proximal tubule epithelial HK-2 cells, incubated with a high glucose concentration (30 mM). The polyphenols significantly inhibited DPP4 activity without altering DPP4 protein concentration, and a similar effect was observed when linagliptin was administered. These results demonstrate that DPP4 inhibition exerted by linagliptin or *H. sabdariffa* polyphenols, mitigates specific markers of insulin resistance (Ser307 phosphorylation of IRS-1) and epithelial-to-mesenchymal transition (vimentin), both of which contribute to the progressive loss of renal function that is characteristic of advanced T2DM. It is highly interesting that DPP4 inhibition modulates the physiological changes that lead to loss of renal function, and it is tempting to suggest that GLP1 signaling was directly responsible, but because of the broad substrate specificity of DPP4, it is not possible to unambiguously confirm it with this data. Complementary experiments on diabetic Sprague–Dawley rats showed that *H. sabdariffa* polyphenols (tube-fed 200 mg/kg·BW) exerted similar changes in vivo, suggesting polyphenol-mediated DPP4 inhibition can preserve renal function in diabetic organisms, similarly to linagliptin.

The effects of sorghum (*Sorghum bicolor* L. Moench) consumption were determined in healthy adult volunteers (20 male, 20 female) by Stefoska-Needham et al. [37]. The volunteers consumed a breakfast of 50 g of red, white, and brown-grained sorghum prepared as biscuits, and compared with a similar amount of wheat biscuits as a control. Serum analysis of enteroendocrine hormones indicated that the area under the curve (AUC) for GLP1, GIP, and PYY was significantly higher as compared to the control. In addition, significantly higher satiety was reported by the participants who consumed sorghum biscuits. The biscuits prepared from the sorghum with the highest polyphenolic content (red sorghum) also exerted the strongest changes on hormone secretion. However, as the authors point out, they were unable to determine the exact mechanisms on appetite suppression and digestive responses during the experimental period, and therefore proposed additional experiments. A possible DPP4 inhibition may have been exerted by the sorghum polyphenols, because the concentrations of GLP1, GIP, and PYY were increased, and as previously stated, they are DPP4 substrates, but this was not experimentally corroborated.

### 3.2. Effects of Polyphenols on Insulin

Anhê et al. [38] used a commercial polyphenol-rich cranberry extract, and administered it by oral gavage to 36 C57BL/6J mice (200 mg/kg·BW), which were maintained on a high fat/sucrose diet, during an eight-week period. The treatment decreased fasting insulinemia, suggesting that the extract increased insulin sensitivity. This was subsequently validated by an oral glucose tolerance test, in which decreased insulin and C-peptide concentration was confirmed. These and other effects were partially attributed to changes in gut microbiota, specifically, an increased population of Akkermansia spp. The presence of the related Akkermasia muciniphila has been correlated with decreased obesity and insulin resistance, through mechanisms that may involve fermentation products, interaction with other bacteria and others [39]. This suggests that polyphenols may exert an indirect effect on insulin secretion by modulation of gut microbiota.

Qin et al. [40] demonstrated the effects of a polyphenol-rich aqueous cinnamon extract (10 and 100 μg/mL) on Wistar rat enterocytes. The treatments increased mRNA expression of the IR, IRS-1, IRS-2, phosphatidylinositol 3-kinase (PI3K) and Akt1; mRNA expression of the phosphatase and tensin homolog (Pten) was decreased. Altogether, these changes in gene expression suggest insulin sensitivity on the enterocytes was increased by the cinnamon polyphenols. An HPLC-MS analysis of the extracts showed that type-A polyphenol trimers and tetramers were present, and may be the active components of the cinnamon extract.

Yamashita et al. [41] extracted procyanidin oligomers from cacao liquor and acutely administered them individually to male ICR (Institute of Cancer Research) mice (10 μg/kg·BW, p.o.). The animals were sacrificed an hour after the treatment, and ELISA analyses were used to quantify GLP1 and insulin. Among the studied compounds (epicatechin, procyanidin B2, procyanidin C1, and cinnamtannin A2), cinnamtannin A2 increased GLP1 and insulin concentration, and was the most bioactive overall. The same compound also exerted significant changes on skeletal (soleus) muscle, by increasing IR-β and IRS-1 phosphorylation. It was later discarded that IRS-1 phosphorylation was related to the JAK-STAT (Janus Kinase-Signal Transducer and Activator of Transcription) pathway, supporting the hypothesis that the IR was responsible for IRS-1 phosphorylation and downstream signaling. All compounds present in the cacao liquor samples are epicatechin oligomers, for example, procyanidin B1 is an epicatechin dimer, procyanidin C1 is an epicatechin trimer, and cinnamtannin A2 is an epicatechin tetramer. The evidence supports the hypothesis that the length of the procyanidin is key to the insulin-sensitizing results, as demonstrated by a lack of significant effects by the epicatechin monomer, dimer and trimer.

The effects of cocoa flavanols were analyzed by Dorenkott et al. [42] on male C57BL/6J mice that were fed a high fat diet for 12 weeks. The diets of the animals were supplemented with either cocoa flavanol extract, or the flavanol fraction enriched with monomeric, oligomeric, or polymeric procyanidins (262 mg/kg of diet) and compared to a low fat diet group. The high fat diet impaired the 12 h fasting glycemia and the AUC of an oral glucose tolerance test, but the oligomeric fraction was able to exert an intermediate improvement in both parameters. A more noticeable and significant effect was evident on a 4 h fasting glycemia and insulin tolerance test, where the oligomeric fraction maintained similar values to the low fat control group, and significantly decreased both, as compared to the untreated high fat group. Fasting hyperinsulinemia was recorded on the untreated group, which was significantly mitigated by the monomeric, oligomeric and polymeric cocoa procyanidins. According to these data, and similar to the findings of Yamashita et al. [41], procyanidin length determines its effectiveness as an insulin secretagogue and sensitizer. The most overall effects were exerted by the oligomeric fraction, the majority of which were trimers to hexamers (~63% of the extract), followed by dimers (~30% of the extract).

Syringic acid is a simple phenolic (benzoic) acid, whose antidiabetic potential was analyzed by Muthukumaran et al. [43] in male alloxan-induced diabetic Wistar rats. Syringic acid (50 mg/kg·BW) was administered for a 30-day period through an intragastric tube. As expected, the diabetic rats showed hyperglycemia and hypoinsulinemia, but the syringic acid treatment reverted both parameters to values almost identical to those of healthy animals. The main mechanism of action of syringic acid was as insulin secretagogue, which was corroborated by a significant increase in the concentration of C-peptide. An insulin sensitizing effect was not analyzed, thus, it cannot be confirmed.

Mangoes (*Mangifera indica* L.) contain phenolic acids as part of their bioactive components, among which is syringic acid. Evans et al. [44] administered 10 g/day of freeze-dried edible mango pulp to 20 overweight adults (11 male, nine female) for 12 weeks. After the experimental period, all subjects had significantly reduced glycemia, but only the male participants had significant increases in insulinemia. No changes in insulin sensitivity were recorded, and the exact cause for the male/female differences was not determined. The authors suggested that mango consumption may have effects on daily glucose/insulin, rather than long-term effects, but this was also not conclusively verified.

Apples are another polyphenol-rich fruit with potential antidiabetic actions. Manzano et al. [45] administered an apple polyphenol extract to obese male Zucker rats, in order to determine its acute and chronic effects on insulin sensitivity. The acute effect (150 mg/kg·BW p.o. of apple polyphenol extract) showed a significantly reduced postprandial glucose response after consumption of apple phenolics, but not on insulin secretion. The chronic effect was determined after five weeks of polyphenol intake (3 g of extract/kg of diet). No significant changes were determined on fasting glycemia, but the apple polyphenols exerted significant postprandial decreases on glucose and insulin responses. To further investigate the mechanism of action, the authors evaluated the effect of apple polyphenols (0–25 μg/mL) on murine L6-myocytes after an 18 h incubation period, with or without insulin. It was determined that the polyphenols exerted a dose-dependent increase in glucose uptake (mediated through increased GLUT4), and synergy was apparent when insulin and polyphenols were both present. Subsequent experiments also demonstrated that the effects of the phenolics decreased if the cells were treated with a PPARγ inhibitor, and that the phenolics acted nearly identical to rosiglitazone, thus showing that glucose uptake was mediated through PPARγ activation. The exhaustive set of experiments of Manzano et al. provided robust evidence that the insulin-sensitizing effects of apple polyphenols were similar to those of the TZDs, particularly rosiglitazone. However, it can be argued that apple polyphenols may be superior to the pharmaceutical, since they also exerted acute actions after merely 30 min of their ingestion.

Törrönen et al. [46] analyzed the effects of consuming a sucrose load or a sucrose load with berries (blackcurrants (*Ribes nigrum*), bilberries (*Vaccinium myrtillus*), cranberries (*Vaccinium oxycoccos*) and strawberries (*Fragaria × ananassa*)) in healthy adult participants (two male, 10 female). The effects of a test meal of 150 g of berry purée with 35 g of sucrose was compared to the same amount of sucrose dissolved in water. A 2 h capillary and venous glucose curve showed that the berries significantly prevented a sharp increase in the first 15 min, and a sharp decrease at 90 min after meal intake. A similar pattern was observed on the serum insulin curve, where the berries prevented pronounced insulin peaks at 15, 90 and 120 min. The AUC for GLP1 was not statistically significant (*p* = 0.05). The main phenolic components reported were anthocyanins, flavonols, phenolic acids, proanthocyanidins, and ellagitannins, totaling approximately 800 mg in the test meal. The results suggested that berry polyphenols modulated insulin secretion and postprandial glycemia by shaping the insulin and glucose curves of the participants.

Olive trees (*Olea europaea* L.) produce edible leaves and fruits, both of which are sources of highly bioactive phenolic compounds, particularly hydroxytyrosol and oleuropein. de Bock et al. [47] administered capsules with olive leaf extract (OLE) or placebo (safflower oil) to overweight middle-aged men for twelve weeks (body mass index, BMI = 28 kg/m^2^), followed by a six-week washout and crossover to the other treatment for an additional 12 weeks. The main components of the extract were oleuropein (51.1 mg) and hydroxytyrosol (9.6 mg). The OLE treatments improved glycemia, insulin sensitivity and β-cell function, as determined by oral glucose curves and the Matsuda insulin sensitivity test [48]. Based on their findings, the authors highlight the dual effect of OLE, which is able to act as an insulin secretagogue and as an insulin sensitizer, something that pharmaceutical treatments are unable to simultaneously replicate. Since the participants were not diabetic, olive phenolics acted in a preventive manner. Considering the overweight middle-aged male phenotype of the participants, which favors the onset of diabetes, the significance of the study is highlighted.

An interventional study performed by Bozzetto et al. [49] analyzed the effects of diets rich in either long chain omega-3 fatty acids, polyphenols, or both, on overweight/obese adults during an eight-week period. Fasting glucose, insulin, insulin secretion, or insulin sensitivity remained unaffected by the treatments. Subsequent oral glucose tolerance tests revealed that the polyphenols were the most bioactive (as compared to the fatty acids), by significantly decreasing the glucose AUC and increasing insulin secretion (analyzed with the homeostasis model assessment method (HOMA)) and sensitivity (analyzed with the quantitative insulin sensitivity check index (QUICKI)). The sources of polyphenols were decaffeinated coffee and tea, dark chocolate, blueberry jam, extra-virgin olive oil, artichokes (*Cynara scolymus var. scolymus*), onions (*Allium cepa* L.), spinach (*Spinacia oleracea* L.), and rocket (*Eruca sativa* Mill.). Synergy was likely exerted between the different classes of polyphenols on the overall insulin metabolism, initially by increasing its secretion and then by promoting peripheral sensitivity to it. Furthermore, the effects were obtained only by ingesting them within food, without the need for additional supplementation, so it is reasonable to consider that similar effects can be replicated with other polyphenol-rich foods available at other geographical locations.

The previously discussed evidence of the antidiabetic effects of polyphenols related to GLP1, DPP4 or insulin are summarized in Table 2.

**Figure 4:**
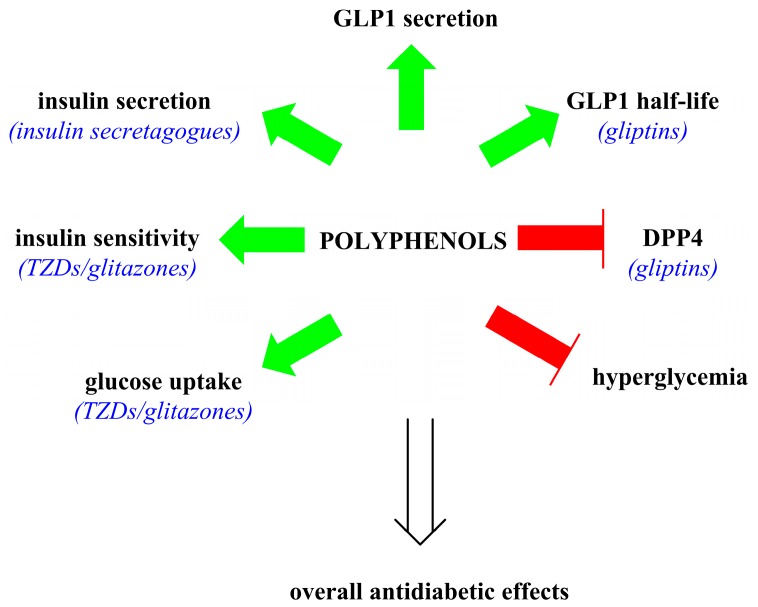
Summary of the overall antidiabetic actions of polyphenols (bold text) that are comparable to pharmaceutical antidiabetics (blue text). Green arrows indicate an increase and a red bar-headed line indicates inhibition.

Figure 4 summarizes the generalized actions of polyphenols as antidiabetic compounds that were discussed in the text.

### 3.3. Targeted Application of Polyphenolics as Antidiabetics

The previously described experimental evidence suggests that various polyphenols favorably modulate the GLP1 and insulin pathways. From the authors’ point of view, there are three possible ways to obtain the most antidiabetic benefits from polyphenols. First, as determined by Bozzetto et al. [49], antidiabetic effects are achievable by means of adequate dietary choices without additional intervention. This implies that regularly consuming polyphenol-rich foods can reduce the risk of diabetes, and epidemiological data is available to back up this hypothesis [50,51]. This option is likely to have the most impact if polyphenol rich foods are consumed from the earliest stages of life, and if these dietary choices are continued through adolescence and adulthood.

Second, consumption of polyphenols in extracts, supplements, functional, or nutraceutical foods and beverages. This alternative may be adequate to increase daily polyphenolic consumption or to compensate for low intake. However, it should also be stated that this may lead to an overdependence on a handful of molecules from a single source, which limits the potential variety of a polyphenol-rich diet (which can also contain other micronutrients), and their benefits may still be nullified by other dietary choices. Another possible disadvantage is a negative drug–nutrient interaction between the polyphenols and other pharmaceuticals that may be co-ingested; the grapefruit effect is one of the most known cases where a food-derived compound has important repercussions for the metabolism of numerous pharmaceuticals [52]. Furthermore, while polyphenols are generally safe when consumed from food, they also have the potential to induce negative side effects if ingested in a concentrated form, either acutely or after a chronic exposure. Thus, a cation must be exerted when consuming them, in order to prevent negative side effects or an overdependence on them.

Third, rational design of antidiabetics based on a polyphenolic structure. This is the most challenging approach because the development and successful introduction of any pharmaceutical compound may require decades of work and vast monetary resources. Drug design based on natural compounds is by no means a new concept, but the structure of polyphenols has not been used as a model to develop antidiabetics. Based on the information discussed in the present work, a successful approach may involve a series of quantitative structure–activity relationship (QSAR) analyses of polyphenols, to improve their already demonstrated activity, and to increase their specificity. Procyanidins are particularly interesting examples, because their length has been tentatively proven to be directly related to their bioactivity as insulin secretagogues and sensitizers. A detailed QSAR analysis and proper molecular modifications of procyanidin oligomers (or others) may lead to a targeted and potentiated effect on L-cells, β-cells, or peripheral tissues, that results in significant improvements of their glucose-lowering abilities.

## 4. Conclusions

The potential antidiabetic actions of various polyphenols were reviewed, and it was found that they are related, at least in part, to an increased secretion of glucagon-like peptide-1 (GLP1) from intestinal L-cells, inhibition of dipeptidyl peptidase-4 (DPP4) that increases GLP1 half-life, increased insulin secretion by (direct or indirect) β-cell stimulation, and increased insulin sensitivity on peripheral tissues (an effect that may be mediated by the PPARγ transcription factor). Their effects and mechanisms of action suggest that they may act in a comparable way to some pharmacological antidiabetics, but further studies and clinical trials are required to support or counter this claim. Their continued study at the basic and clinical levels may eventually yield newer classes of compounds that prevent type-2 diabetes mellitus diabetes or mitigate its detrimental consequences on human health.

## Figures and Tables

**Table 1:** Pharmacological agents to treat type-2 diabetes mellitus (T2DM) that act on glucagon-like peptide-1 (GLP1), insulin or insulin sensitivity.

Class	Mechanism of Action	Main Examples (International Non-Proprietary Name)	Reference
DPP4 inhibitors (gliptins)	Prevent GLP1 hydrolysis	Sitagliptin, vildagliptin, saxagliptin, linagliptin	[21,22]
GLP1R agonists (incretin mimetics)	Mimic GLP1 signaling	Exenatide, liraglutide, lixisenatide, albiglutide, dulaglutide	[23]
Insulin secretagogues (sulfonylureas)	Stimulate insulin secretion	Glibenclamide, glimepiride	[24,25]
Insulin secretagogues (glinides)		Repaglinide, nateglinide, mitiglinide	
Insulin sensitizers (thiazolidinediones/glitazones)	Increase insulin sensitivity	Rosiglitazone, pioglitazone	[27]

**Table 2:** Concise summary of the effects of polyphenols from various sources on glucagon-like peptide-1 (GLP1), dipeptidyl peptidase-4 (DPP4) and insulin.

Polyphenol Source	Model	Effect on GLP1	Effect on DPP4	Effect on Insulin	Reference
Sweet potato ethanolic extract	Murine GLUTag cells	↑ secretion			[28]
Sweet potato ethanolic extract	Sprague–Dawley rats	↑ secretion			[28]
Coffee extract	Human NCI-H716 cells	Dose-related ↑ secretion			[29]
Coffee extract	C57BL/6J mice	↑ secretion			[29]
Cocoa products			Inhibit		[31]
Grape seed extract		↑ secretion	Inhibit	↑ secretion	
Grape seed extract	STC-1 cells	↓ secretion			[33]
Grape seed extract	Wistar rats	↑ secretion		↑ secretion	[34]
*Hibiscus sabdariffa*	HK-2 cells		Inhibit		[36]
*Hibiscus sabdariffa*	Sprague–Dawley rats		Inhibit		[36]
Sorghum	Healthy adults	↑ GLP1 area under the curve			[37]
Cranberry extract	C57BL/6J mice			↓ fasting insulinemia	[38]
Cinnamon extract	Wistar rat enterocytes			↑ sensitivity	[40]
Procyanidin oligomers from cacao liquor	ICR mice	↑ secretion		↑ secretion	[41]
Cocoa flavanols	C57BL/6J mice			↓ fasting insulinemia	[42]
Syringic acid	Diabetic Wistar rats			Normalized glycemia and insulinemia	[43]
Mango	Overweight adults			↑ insulinemia in males	[44]
Apple polyphenol extract	Obese Zucker rats			No effect on postprandial insulinemia (acute effect)	[45]
Apple polyphenol extract	Obese Zucker rats			↑ postprandial insulin response (chronic effect)	[45]
Apple polyphenol extract	Murine L6-myocytes			Polyphenol/insulin synergy on glucose uptake	[45]
Blackcurrants, bilberries, cranberries, and strawberries	Healthy adults			Prevented insulin and glucose peaks	[46]
Olive leaf extract	Overweight men			↑ sensitivity	[47]
Dietary intervention with polyphenol-rich foods	Overweight adults			↑ secretion and sensitivity	[49]

↑: increases; ↓: decreases. STC: secretin tumor cells; ICR: Institute of Cancer Research.
